# Stages of endometriosis: Does it affect oocyte quality, embryo development and fertilization rate?

**DOI:** 10.5935/1518-0557.20220051

**Published:** 2022

**Authors:** Sedighe Esmaeilzadeh, Mahsa Ghorbani, Maryam Abdolahzadeh, Mohammad Chehrazi, Sayed Gholamali Jorsaraei, Parvaneh Mirabi

**Affiliations:** 1 Infertility and Reproductive Health Research Center. Health Research Institute, Babol University of Medical Sciences, Babol, Iran; 2 Department of Biostatistics and Epidemiology, School of Medicine, Babol University of Medical Sciences, Babol, Iran

**Keywords:** endometriosis, embryo quality, ovarian response, intracytoplasmic sperm injection, oocyte competence

## Abstract

**Objective:**

To investigate the effect of endometriosis and its different stages over Intracytoplasmic Sperm Injection (ICSI) outcomes among infertile women without previous history of ovarian surgery.

**Methods:**

A total of 440 women enrolled in ICSI cycles were recruited and divided into two groups: endometriosis (n=220) and control group (n=220). Endometriosis patients without previous surgical treatment and with diagnostic laparoscopy were further stratified based on disease stage. Clinical and laboratory parameters, ovarian reserve markers, the number and quality of oocytes and embryos and fertilization rate were analyzed and compared among the various severity grades of endometriosis and the control group.

**Results:**

Patients with advanced endometriosis had significantly fewer retrieved oocytes with small effect size (*p*<0.001, η^2^=0.04), lower metaphase II oocytes (*p*<0.001, η^2^=0.09) and fewer total numbers of embryos (*p*<0.001, η^2^=0.11) compared with less severe disease or women with tubal factor infertility. The fertilization rate in women with severe endometriosis was similar to that of the control group and in those with minimal/mild endometriosis (*p*=0.187).

**Conclusions:**

Severe endometriosis negatively affects ovarian response, oocyte quality and embryos. However, fertilization rate is not different among the various stages of endometriosis.

## INTRODUCTION

Endometriosis is an enigmatic disorder affecting 10-15% of females of reproductive age. It is estimated that up to 40% of women undergoing laparoscopic investigation for infertility have endometriosis (Yilmaz *et al*., 2021). Endometrioma may be present in 17-44% of women diagnosed with endometriosis (Alshehre *et al*., 2021; Hoyle & Puckett, 2022). Endometriosis is a debilitating disease and has a considerable economic burden on patients and the society (Esmaeilzadeh *et al*., 2015; Parasar *et al*., 2017).

Classification of endometriosis has been established by the American Society for Reproductive Medicine (ASRM), and it can be categorized into four stages: I (minimal), II (mild), III (moderate), and stage IV (severe) (Mirabi *et al*., 2019). More advanced stages may be deeply invasive and present as endometrioma that can lead to specific complications, such as decreased ovarian reserve in these patients (Hoyle & Puckett, 2022).

Still, endometriosis is frequently reported as the underlying etiology of subfertility, so assisted reproductive technology (ART) is the common choice for patients with endometriosis-associated sterility who wish to conceive. Moderate and severe stages of endometriosis usually require *in vitro* fertilization (IVF) (Alshehre *et al*., 2021). Whether endometriosis contributes to infertility has long been debated, and underlying mechanisms resulting from the presence of disease and classified by stage possibly affecting fertility potential are poorly known, although inflammation and reactive oxygen species are believed to contribute significantly (Rogers *et al*., 2017).

Research findings showed that endometriosis patients may have endocrine and ovulatory disorders, including impaired folliculogenesis compromised granulosa cell and follicle immune homeostasis, premature luteinizing hormone (LH) surges and luteal phase defects (Hamdan *et al*., 2015; González-Comadran *et al*., 2017).

Some investigators strongly emphasize that oocytes from endometriosis patients have reduced competence, and high stages of endometriosis consistently lead to reduced oocyte yield (Horton *et al*., 2019). Clinical IVF/ICSI studies have investigated antral follicle count, number and quality of retrieved oocytes and embryos, cycle cancellation and fertilization rates in endometriosis patients have either not found significant differences (Hamdan *et al*., 2015; González-Comadran *et al*., 2017) or detected meaningfully diminished ovarian reserve, oocyte yield and number of mature oocytes (Xu *et al*., 2015; Horton *et al*., 2019; Orazov *et al*., 2019; Alshehre *et al*., 2021).

A recent retrospective cohort study stated that the number of antral follicle count (AFC) and mature oocytes were significantly lower in infertile women with endometrioma, whereas numbers of embryos achieved, clinical pregnancy rates and live birth rates were similar between endometriosis patients and control groups (Yilmaz *et al*., 2021). On the other hand, some studies have reported an inverse relationship between different stages of endometriosis and ART outcome. They stated that advanced endometriosis has a deleterious and sustained effect on ovarian reserve and fertility parameters (Kuivasaari *et al*., 2005; Pop-Trajkovic *et al*., 2014; Orazov *et al*., 2019).

Li *et al*. (2020) showed a negative effect of advanced endometriosis on cumulative clinical pregnancy per oocyte retrieval cycle, also a systematic review and meta-analysis of the available comparative and observational data suggested that a progressive reduction in ART outcomes has been shown in endometriosis-affected patients with increasing disease severity (Horton *et al*., 2019).

This finding is contrary to previous systematic reviews and meta-analyses, which stated that there was no relevant difference in the chance of achieving clinical pregnancy and live birth following ART when comparing Stage-III/IV with Stage-I/II endometriosis (Barbosa *et al*., 2014). However, the quality of most of the studies in this systematic review has been very low to moderate. Anyway, reports evaluating the impact of endometriosis and different stages of it on the outcomes of IVF/ICSI seemed controversial, in relation to the focus on different specific outcomes. As yet, several questions remain unanswered. There is no robust data to recognize the plausible negative impact of various stages of endometriosis on the main outcomes of ART (number of AFC, oocyte competence, embryo development and fertilization rate), especially for limited (Stage-I/II) disease. For these reasons this retrospective study was conducted to investigate whether the presence and/or the severity of endometriosis affects ICSI outcomes, including oocyte quality, fertilization rate and embryo quality.

## MATERIALS AND METHODS

This investigation was a 1:1 retrospective cohort study of patients undergoing first-attempt ICSI treatment at the Fatemezahra Infertility and Reproductive Health Research center, Babol, Iran. The study performed in compliance with the Institutional Review Board at Babol University of Medical Sciences (NO.IR.MUBABOL.HRI.REC.1398.296) between November 2019 and December 2020 - STROBE (Strengthening the Reporting of Observational studies in Epidemiology) guidelines for observational studies were followed to conduct this study.

All data were obtained from databank and medical records of infertile patients who referred to our infertility center. This center is one of the best equipped diagnostic and therapeutic infertility centers, and it is a referral center for infertility problems in the north of Iran.

### Selection of participants and data collection

The eligibility criteria were <40 years of age, first ICSI, normal sperm in the male and all had previously undergone diagnostic laparoscopy and transvaginal ultrasound using (5 MHz probe Fokuda, Japan). The exclusion criteria were polycystic ovaries, immunological disease, uterine abnormalities, previous history of ovarian surgery, premature ovarian failure and severe male factor according to WHO criteria. Infertile women with laparoscopic and ultrasound confirmation of endometriosis based on the inclusion criteria were selected as the study group, and women with tubal factor diagnosis or unexplained infertility were included as the control group. Endometriosis was staged according to the ASRM 1996 classification (based on laparoscopy) (Mirabi *et al*., 2019).

### Oocyte retrieval

To stimulate the follicles, ovulation induction protocol using agonists GnRH (Superfact, Aventis Pharma Deutschland, Germany) was started in the mid-luteal phase of the cycle (21-23 of the cycle). For initiating, a thin dense endometrium < 4 mm, and the absence of any growing follicle > 6 mm should be visualized in the transvaginal ultrasonography (TVS). If the endometrial line was thin in TVS (< 5mm) 14 days after the agonist started, we did controlled ovarian hyperstimulation (COH) using recombinant human follicle-stimulating hormone (Cinnal-f, Cinagen-Iran ). Cinnal-f dose was chosen for each patient on the basis of age, day 3 FSH and AMH level and AFC if the endometrial line was not thin in the TVS, superfact continued for two following weeks. When the endometrial line reached <5mm, the stimulation was initiated.

If not, the cycle was canceled, but the patient did not drop out of the study. Follicle’s monitoring was performed 7 or 8 days later and every 2 to 3 days through TVS to trace the growing follicles. Cinnal-f was added according to ovarian response. Human Chorionic Gonadotropin 10,000 IU (HCG, Daroupakhsh, Iran) was administrated when at least two oocytes >16 mm were seen. Oocytes were retrieved through TVS-guided 34-36 hr following HCG trigger.

Collected oocytes were classified into Metaphase II (MII), metaphase I (MI) and Germinal vesicles (GV). Embryos were graded morphologically.

Embryos with little or no fragmentation and a zona pellucida not extremely thick or dark in appearance were classified as Grade A, embryos with equally-sized blastomeres, minor cytoplasmic fragmentation covering ≤10% of the embryo surface Grade-B and blastomeres of distinctly unequal size and moderate-to-significant cytoplasmic fragmentation covering >10% of the embryo surface were Grade C (Mirabi *et al*., 2017). One or two embryos were transferred per transfer cycle. In each patient, 2 grade-A embryos were transferred on day 2 or 3.

Baseline characteristics of participants, ovarian reserve biomarkers (AMH, FSH, and AFC), the number and quality of oocytes and embryos and fertilization rate were analyzed and compared between various severity grades of endometriosis and control group.

### Statistical analysis

All statistical analyses were performed using a software (SPSS 21.0 version). Kolmogorov-Smirnov test was used to evaluate the normality of variable distribution. The Continuous variables with normal distribution were expressed as the mean±SD or as percentages when required. Comparison of demographic and clinical characteristics in the various severity grades of endometriosis and the control group were performed with one-way analysis of variance (ANOVA), with Bonferroni adjustment. The mean difference (MD) and their 95% confidence interval (CI) were calculated for the studied outcomes. Results were also compared between patients with Stage I/II and Stage III/IV endometriosis and the control group. Effect sizes were expressed as Eta Squared (*η*^2^) to show the relative magnitude of the differences between means (Small = 0.01. Moderate = 0.06 or Large = 0.14) (Lakens, 2013).

## RESULTS

**Figure 1:**
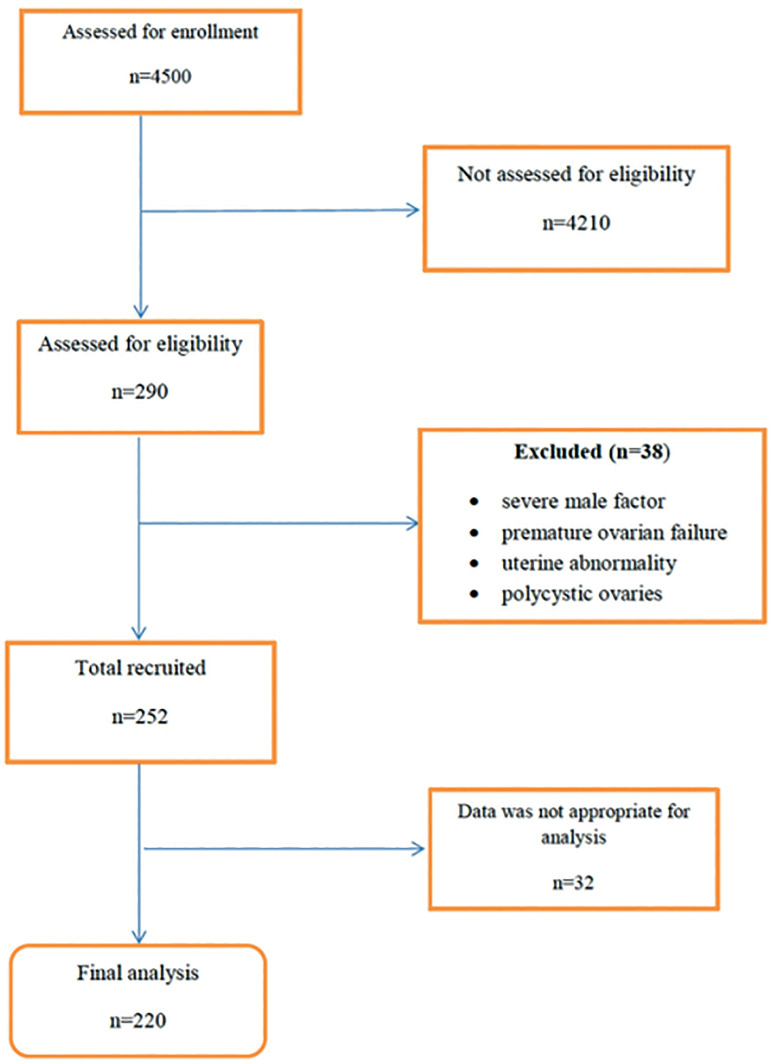
Flow of participants through the study.

Two hundred and ninety women were diagnosed with endometriosis. Thirty-eight were excluded for different reasons (10 patients had been diagnosed with severe male factor; 2 with premature ovarian failure; 8 with uterine abnormality and 18 patients had polycystic ovaries) (Fig. 1). The data of 32 patients were not appropriate for analysis, therefore; they were also excluded from the study.

Of the 220 endometriosis patients, 48 (21.8%) had ASRM stages I endometriosis, 76 (34.5%) stage II, 53 (24%) stage III and 43 (19.5%) stage IV. Two hundred and twenty women with unexplained infertility or laparoscopically diagnosed tubal factor infertility (without any evidence of endometriosis) were included as the control group.

The mean age was 32.4**±**5.16 years in the endometriosis group and 31.44±5.64 in the control group (*p*=0.25). The mean **±**SD body mass index of the endometriosis participants was 25.50±3.0kg/m^2^ *versus* 25.95±4.35. Therefore, most of the participants were overweight, however, differences between groups were not statistically significant. Therefore, if BMI is a factor influencing ART outcomes, this effect is the same between the groups (*p*=0.26). Dyspareunia and infertility duration were higher in the women diagnosed with endometriosis than controls, and this difference was significant. There were no statistically significant differences in terms of dysmenorrhea, type of infertility (primary or secondary) and abortion between groups (Table 1).

**Table 1:** Characteristics and hormonal assay results of study population.

Variables	Endometriosis n=220	Without Endometriosis n=220	*p*-value
**Age (years)**	32.4±5.16	31.44±5.64	0.25
**BMI (kg/m^2^)**	25.50±3.0	25.95±4.35	0.26
**Duration of infertility (years)**	2.03±0.73	2.17±0.73	0.03
**Type of infertility n (%)** Primary secondary	154 (70.3) 65 (29.7)	160 (72.4) 61 (27.6)	0.35
**Dysmenorrhea**	147 (67.1)	145 (65.6)	0.40
**Dyspareunia**	33 (15.1)	14 (6.3)	0.002
**Abortion**	40 (18.2)	38 (17.3)	0.53

Data are expressed as mean ± SD or n (%).

The plasma levels of FSH and prolactin were significantly higher in the endometriosis compared with tubal and unexplained groups (*p*<0.001); however, there no significant differences between groups according to the levels of AMH; also, the total duration of gonadotropin stimulation was not statistically significant between the groups (Table 2).

**Table 2:** Hormonal assay results and ICSI outcomes in endometriosis and control groups.

*Covariate*	*mean±SD*		*p*-value^*^	Mean difference^**^	95% Confidence Interval of the Difference	
	Endometriosis n=220	control n=220			Lower	*Upper*
**FSH (mIU/L)**	8.03 ±6.26	6.11 ±2.21	**<0.001**	1.92	1.03	2.80
**LH (mIU/L)**	6.21 ±6.16	5.33 ±2.51	0.06	0.87	-0.04	1.79
**Prolactin (ng/mL)**	18.05 ±12.22	14.78 ±6.19	**0.001**	3.27	1.32	5.21
**AMH**	3.30 ±6.84	3.17 ±2.97	0.89	0.12	-1.74	1.99
**Duration of stimulation (days)**	10.5 ±3.1	9.22 ±1.7	0.05	1.28	092	2.90
**Total oocytes**	4.80 ±4.5	6.92 ±5.31	**<0.001**	-2.12	3.15	1.08
**Metaphase II oocytes**	1.85 ±3.63	4.02± 4	**<0.001**	-2.21	-3	1.42
**Total embryos**	2.10 ±2.85	3 ±2.98	**0.001**	-0.90	-1.45	0.36
**Good quality embryos**	1 ±1.77	2.02 ±2.2	**<0.001**	-1.02	-1.41	0.64
**Fertilization rate**	0.61 ±0.52	0.65 ±0.57	0.49	-0.03	-014	0.06

Data are expressed as mean ± SD.

On the other hand, the oscillations of FSH and prolactin levels varied in relation to the stage of endometriosis progression. As to ANOVA test, FSH and prolactin levels were significantly higher in stages III/IV compared with controls (*p*<0.001) (Table 3).

**Table 3:** ICSI data and clinical outcome of patients with different stages of endometriosis and control groups.

*Covariate*	mean±SD			*p*-value	Eta Squared (η^2^)
	Endometriosis I/II n=124	Endometriosis III/IV n=96	Control n=220		
**FSH (mIU/L)**	7.82 ±4.92	8.27 ±7.74	6.13 ±2.20	<0.001	0.04
**AMH(ng/mL)**	3.57 ±8.35	2.94 ±2.6	3.12 ±2.97	0.88	0.003
**Prolactin(ng/mL)**	17.17 ±11.62	19.38 ±12.93	14.72 ±6.14	<0.001	0.05
**Total oocytes**	5.37 ±4.45	4.90 ±4.4	6.92 ±5.32	<0.001	0.04
**Metaphase II oocytes**	2.52 ±3.53	2.07 ±3.04	4.60 ±4.6	<0.001	0.09
**Total Embryos**	2.41 ±3.04	1.68 ±2.5	3.01 ±2.9	0.001	0.05
**Good quality embryos**	1.24 ±2	0.6 ±1.3	2.03 ±2.20	<0.001	0.11
**Fertilization rate**	0.66 ±0.45	0.54±0.59	0.65 ±0.57	0.18	0.01

Data are expressed as mean ± SD.

Despite similar levels of serum AMH between the groups, fewer oocytes in women with infertility associated with endometriosis were retrieved than in patients in the control group *(p*<0.001). Also, we found a statistically significant decrease in the number of mature oocytes (MII) in endometriosis patients, compared with the control group *(p*<0.001).

ANOVA-repeated measures revealed that patients with advanced endometriosis (III/IV) had significantly fewer retrieved oocytes with small effect size (*p*<0.001, η^2^=0.04), lower metaphase II *oocytes* (*p*<0.001, η^2^=0.09) and fewer total numbers of embryos (*p*<0.001, η^2^=0.11) compared with less severe disease or women with tubal factor infertility. The effect size was medium. The fertilization rate in women with severe endometriosis was similar to that of the control group and in those with minimal /mild endometriosis (*p*=0.187).

Pregnancy rates were significantly different in various stages of endometriosis, as patients with severe endometriosis had a lower pregnancy rate compared to less severe disease (*p*=0.02).

## DISCUSSION

Over the *past two decades*, the detrimental influence of endometriosis on ART outcomes has been debated in the literature. There is controversy regarding the impact of different stages of endometriosis on assisted reproductive technique outcomes (Yilmaz *et al*., 2021).

According to our data, advanced endometriosis had a negative influence on ART outcomes. In the current study, the number of retrieved oocytes, number of MII oocytes and good-quality embryos were decreased in relation to the endometriosis progression. Though we didn’t find considerable differences in the requirement of gonadotrophin with increasing severity of endometriosis.

Elevated prolactin levels were also detected in women with endometriosis, irrespective of the stage of the disease. This study supports evidence from previous observations by Mirabi *et al.* (2019) and Esmaeilzadeh *et al*. (2015), who reported prolactin levels act as a probable prognostic biomarker to detect endometriosis stages III/IV *vs*. I/. The true mechanisms of action of hyperprolactinemia in patients with endometriosis are still not fully understood. However, studies only suggested prolactin concentration progressively increased from stage I to IV (Mirabi *et al*., 2019).

Almost all aspects of ART are negatively influenced by severe endometriosis, from the ovarian response during gonadotropin stimulation to pregnancy. The only exception was the fertilization rate. These results are consistent with data obtained in a systematic review and meta-analysis of 8 published studies. They found that severe endometriosis can significantly reduce the total number of oocytes, MII oocytes retrieved and total high-quality embryos; however, it does not seem to adversely impact fertilization rates (Alshehre *et al*., 2021). Also, Li *et al.* (2020) revealed that despite the number of mature oocytes and viable embryos are meaningfully lower; there were no statistically significant differences between advanced endometriosis and the comparison group with respect to fertilization rate and pregnancy outcomes. Kuivasaari *et al*. (2005) suggested women with advanced endometriosis had worse IVF/ICSI results including fertilization and pregnancy rates compared with women with milder forms of endometriosis or women with tubal infertility.

In addition, Barnhart *et al*. (2002) found detrimental effects of advanced endometriosis on developing follicles, oocyte quality and embryo yield, also they reported lower pregnancy and live birth rates compared to minimal and mild endometriosis.

Although in our study the amount of AMH was not meaningfully different between the groups, the higher levels of FSH in some patients and a diminished number of total and mature oocytes in advanced endometriosis compared with less severe endometriosis enables us to speculate that progression of disease per se is associated with reduced ovarian reserve, but this does not translate into better fertilization rates, which were the same as in women with the most severe forms of disease compared to women with milder forms of endometriosis or other causes of infertility.

Previous studies have suggested that a considerable increase in fertilization failure in IVF is much rather due to the sperm-egg interaction in advanced endometriosis. It could also be related to a lower rate of MII oocytes (Shebl *et al*., 2017). Also, the peritoneal fluid of endometriosis patients may influence sperm-binding ability and sperm motility. Hence, fertility rates are generally reduced. That’s the reason why ART scientists recommend that IVF should not be the first treatment of choice in advanced endometriosis (Hull *et al*., 1998; Shebl *et al*., 2017; Li *et al*., 2020).

All patients in the current study underwent ICSI treatment; thus, fertilization rates were the same in advanced endometriosis compared to mild and moderate grades.

Patients with advanced stages of endometriosis usually receive ovarian surgery, although post-operative having of ovarian reserve deterioration, postulated by clinical evidence (Chiang *et al*., 2015).

Despite the plethora of studies on endometriosis, it is not easy to draw any *definite conclusions* due to the methodological heterogeneity, selection bias of studies, differences in the surgical methods, selected outcomes and low sample size of studies. Although the women included in our study had no previous history of ovarian cyst excision or resection, we found that advanced stage endometriosis *adversely affects* ovarian response and oocyte performance. In fact diminished ovarian reserve is diagnosed as the reduced capacity of the ovaries to create oocytes in both quantity and quality (Zangmo *et al*., 2016). *Most studies (*Aboulghar *et al*., 2003) on severe *endometriosis* usually focus on patients with a previous endometriosis surgery and concluded that patients with a history of previous surgery showed lower responses to gonadotropin stimulation. Also, they had less fertilization rates. Hence; there is still uncertainty on the impact of various stages of endometriosis on IVF-ICSI outcomes.

Due to the lack of surgical history in our participants, it seems that advanced endometriosis without surgical intervention may be one of the critical factors that have a negative effect on ovarian reserve and ovarian response to stimulation. In line with our findings, in a case-control study, Hock *et al*. (2001) found diminished ovarian reserve in stage III/IV endometriosis and concluded this is in accordance with progressive loss of ovarian reserve in patients with advanced endometriosis independent of age. Maneschi *et al.* (1993) also reported a reduced number of developing follicles and vascular activity before any operation among infertile women with severe endometriosis, suggesting that the disease may be detriment to the ovary. However, in most studies fertilization rate was not impaired.

Contrary to our results and aforementioned studies, Pop-Trajkovic *et al*. (2014) reported that fertilization rates in women with severe grades of endometriosis was higher than in those with minimal/mild endometriosis. We do not know *the reason* of *higher fertilization rates among stage* (III/IV) endometriosis patients in that *study*. This observation may support the hypothesis that the burned-out lesions in women with advanced endometriosis is inactive and causes adhesions in the pelvis, while a milder form of endometriosis is associated with active endometrial glands (Pop-Trajkovic *et al.*, 2014).

On the other hand, several cofactors, such as age, BMI and ovarian reserve may be responsible for the poor IVF/ICSI outcomes in these patients. Based on the literature, the impact of diminished ovarian reserve becomes more pronounced in patients of older age whose declining egg quality is associated with higher embryo aneuploidy (Ata *et al*., 2019; Pirtea & Ayoubi, 2020). Women in an aforementioned study were younger than our participants, and most of them had normal weight, while our patients were older and half of them were overweight - which may be one reason for the observed higher rates of fertilization.

Most importantly, patients with advanced stages of endometriosis had meaningfully lower viable embryo rates per oocyte retrieved, which suggest the lack of oocyte competence. Additionally, Patients with severe endometriosis had a lower pregnancy rate compared to less severe diseases which is in agreement with other studies (Kuivasaari *et al.*, 2005). However, effect size was small and it was meaningless. A systematic review and meta-analysis demonstrated that clinical pregnancy rates are not relevantly different between patients with advanced endometriosis and those with milder forms of endometriosis. Nevertheless, despite the very large number of included studies and the higher risk of bias in observational studies, they were considered of very low quality and their results were inconsistent (Barbosa *et al*., 2014).

### Strength and Limitation

One of the strengths of this study was that all of the participants had diagnostic laparoscopy and the severity of the disease was determined, which reduced the risk of misclassification. Since the stage of endometriosis, was reported in the data bank of the infertility center, the impact of various stages of endometriosis on ART outcomes was completely ascertained from this analysis. The present study has a few limitations such as our study was a single-center analysis and it had a retrospective design.

## CONCLUSION

Based on the results of this study, we suggest that the high stage of endometriosis may damage ovarian reserve, and it has a detrimental effect on ovarian response during gonadotropin stimulation. We found that patients with severe endometriosis have a trend toward worse outcomes, however; lower ovarian reserves are not associated with lower fertilization and pregnancy rates. Moreover, fertility parameters and reproductive outcomes of stages III/IV and stage I/II endometriosis seem to be comparable. This finding may help clinicians find appropriate advanced endometriosis management.
